# Seismic Performance Assessment of a Moment-Resisting Frame Steel Warehouse Provided with Overhead Crane

**DOI:** 10.3390/ma16072815

**Published:** 2023-03-31

**Authors:** Nicolás Lisperguier, Álvaro López, Juan C. Vielma

**Affiliations:** School of Civil Engineering, Pontificia Universidad Católica de Valparaíso, Valparaiso 2340000, Chile; nicolas.lisperguier.f@mail.pucv.cl (N.L.); alvaro.lopez@pucv.cl (Á.L.)

**Keywords:** special moment resisting frame, pushover analysis, time-history analysis, nonlinear behavior, N2 method

## Abstract

The purpose of this study is to analyze the nonlinear behavior of a steel warehouse structured by moment-resistant frames, which utilizes an overhead crane on its interior brackets and as an external load of the weight of the lining panels. The analysis methods used are (i) pushover analysis, which consists of applying an incremental force in the transverse and longitudinal direction to obtain the capacity curve of the structure; (ii) time-history analysis, in which different records of destructive earthquakes that occurred in Chile are used in order to analyze the response of the structure to these loads. The results indicate that the transverse direction is more ductile than the *Y* direction of the structure within the pushover and time-history methods but not using the N2 method. It is also found that most of the columns are within the life safety and collapse prevention criteria. It is concluded that most of the analyses agree with each other and with what is expected, except for the N2 method, which contradicts the results of the time-history analysis, so the N2 method would not be suitable for this type of structure. In addition, it has been determined that the overhead crane loads do not substantially affect the seismic performance of the warehouse.

## 1. Introduction

Structures with moment-resistant steel frames are widely used, especially for industrial buildings, due to their high resistance to destructive seismic movements (such as those in Chile) [1], thanks mainly to their adequate energy dissipation capacity and the ductility that the structure can achieve. The energy dissipation provided by special moment-resistant frames occurs when plastic hinges are generated in beams, beam-column joints, and the base of the columns [2]. Various investigations address the behavior of moment-resistant frames and their use within industrial structures [3,4,5], most reaching the same conclusion, resulting in an effective type of structuring against seismic loads. However, attention to the overall performance of these structures is necessary because rare-occurrence earthquakes may damage contents or non-structural components [6,7].

The importance of this type of structure is evident, especially in developing countries; however, many small companies require an overhead crane to lift and move loads [8,9,10]. However, these types of structures have received little attention, with only a few studies clarifying their performance against the action of earthquakes [4,11,12]. Although recent works have addressed the seismic response of this type of structure, even proposing procedures to optimize the design of prequalified connections or to specifically study the nonlinear response of such connections, determination of their seismic response still needs to be completed [13,14,15,16].

It is well known that the nonlinear analysis of structures leads to evaluating their response when they exceed the elastic behavior threshold [17,18,19], providing results that make it possible to evaluate the presence of brittle elements or configurations that alter the expected response [20,21,22]. Through this method, the seismic response of structures is characterized under a predetermined lateral load scheme, using a control node to visualize displacements. This analysis method has notable advantages, such as its moderate computational cost and intuitive nature. However, it also has a series of disadvantages, such as the lack of representativeness of its results in the case of irregular structures or structures with characteristics that make it challenging to define lateral loads based on known patterns [23,24,25,26,27]. These analyses are particularly useful for evaluating the capacity of steel structures to carry out pre-and post-seismic strengthening procedures [28].

Nonlinear dynamic analysis allows for incorporating more realistic and representative actions of earthquakes’ effects on structures, non-structural elements, and contents [29,30].

The reference structure of this study is relatively regular in plan and elevation, given its symmetrical geometry that includes the bracing. However, a significant concentrated load induced by the overhead crane can generate mass asymmetry that could alter the dynamic response, as has been studied in some works [31,32,33,34]. The presence of overhead cranes within steel warehouse facilities often affects the complexity of their operations [35] since they involve moving loads that, in some cases, can be decisive in the structural design stage [36,37].

In this work, the analysis of a steel warehouse structure designed according to current code specifications is carried out [38,39,40]. The structure has been sized and detailed considering seismic and wind loads, as well as loads from a bridge crane. The initial objective is to determine if the loads imposed by the bridge crane are determinants in the results of the applied nonlinear analysis, and based on these results, the ASCE 41–17 [41] methodology will be applied to verify if the chosen sections for the structural members adequately respond to the loads imposed by a set of ground motions. The applied procedure could be used to introduce improvements in the design or to retrofit existing structures [42,43].

## 2. Applied Methodology

This research aims to evaluate the seismic performance of a steel warehouse provided with an overhead crane to determine if the weight transmitted by it modifies the structure’s response. The applied methodology starts with designing a simple warehouse with a regular geometry, whose main structure consists of steel frames, designed for a location corresponding to an area with high seismic hazard and considerable wind solicitations. The warehouse is designed and detailed following current normative prescriptions, obtaining a symmetrical structure in which lateral bracing is used to regulate displacements within the margins established for warehouses by building codes.

Simulation of constitutive and geometric nonlinearities is considered to evaluate the seismic performance of the structure resulting from the design. The constitutive nonlinearity requires using representative behavioral models for steel in both static and dynamic ranges once the material’s elastic limit has been exceeded. Geometric nonlinearity considers the effect that large displacements of flexible structures have on the dynamic response, also known as the P-Delta effect. The analyses are carried out first, considering that the structure does not have gravitational loads from the overhead crane. Then, these loads are included in the subsequent analyses.

The seismic evaluation is conducted by applying pseudo-static and dynamic analysis. Pseudo-static analysis, also known as pushover analysis, is applied to determine the demand on the materialized structure through the performance points corresponding to the seismic action defined by the elastic design spectrum. The dynamical analyses are carried out using a set of time-history analyses. The accelerations correspond to selected strong earthquake records of events occurring in the Chilean subduction zone. The results obtained from these analyses make it possible to evaluate the structure’s and its components’ performance by comparing an engineering demand parameter with the thresholds defined in the ASCE SEI 41 [41]. As in the case of pseudo-static analyses, the model with and without loads of the overhead crane has been considered for dynamic analyses.

### 2.1. Model Definition

The structural model used in this study corresponds to a steel shed, which is in the city of Viña del Mar, V region, Chile. The structure falls into the category of a non-essential industrial building. It is a system composed of columns, beams, and bracing of A36-type steel, whose structural element sections are detailed in Table 1.

**Figure 1:**
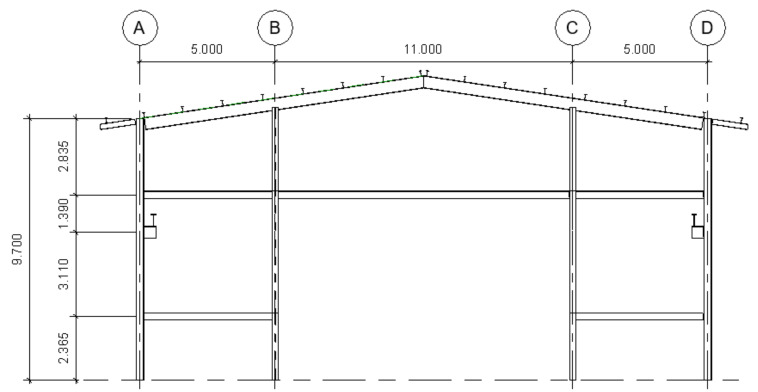
Elevation view of the outer frames (dimensions in m). A, B, C and D are the structural axis.

**Figure 2:**
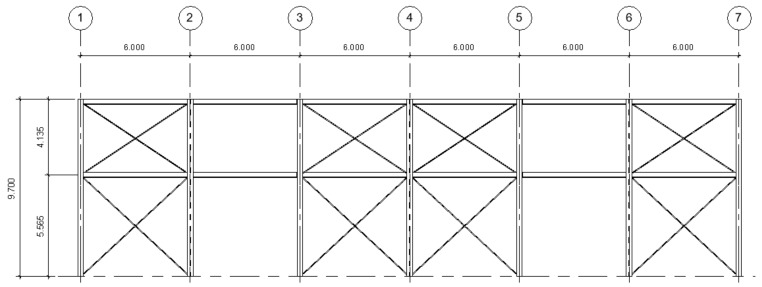
Elevation view of the warehouse (dimensions in m).

**Figure 3:**
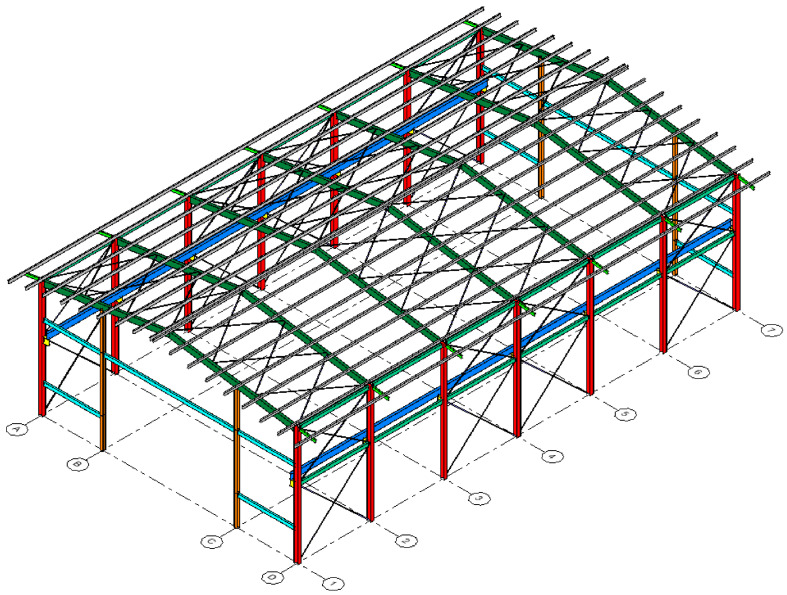
Isometric view of the reference structure. Transverse axis: 1 to 7. Longitudinal axis: A to D.

The structure consists of seven transversal frames with a single span, separated 6 m from center to center. The two outer frames (frames 1 and 7) have a structure corresponding to four columns and three beams. The interior frames (frames 2–6) have only two columns at their base. In contrast, the roof has symmetrical ribs and bracing throughout the entire structure and a slope of 15%, as seen in Figure 1, Figure 2 and Figure 3.

The columns of the entire structure are embedded in one of its ends (base–base). The columns on the sides of all the frames are 9.7 m high from the base, whereas the central columns of frames 1 and 7 are 10.45 m high. In addition, each frame has a pair of corbels on its external columns to support the loads the overhead crane exerts. These corbels are located 5.7 m from the base of the column (as well as the side beams) and have a width of 0.5 m from the junction with the column. Side bracing is also used between the 1–2, 3–4, 4–5, and 6–7 frames, and top bracing between themselves.

The connection of the overhead crane with the structure is carried out through a pair of crane beams oriented along the longitudinal axis of the warehouse. The crane beams are highlighted in deep blue in Figure 3. The crane loads are applied differently, considering that the hoist loaded to its maximum capacity (100 kN) is completely skewed towards one end, generating a maximum load towards the nearby end and a minimum load towards the far end. These loads have been obtained directly from the manufacturer’s instructions [44], considering the overhead crane’s capacity and the warehouse’s free span.

The numerical model is made up of beam-type elements for the beams and columns and bar-type elements for the bracing. Each element in turn has been discretized into fibers, using 200 fibers per cross-section for profiles H and I. The nonlinear behavior of the material of the structural members (A36 steel type) has been modeled using the Menegotto and Pinto model [45] adjusted according to the mechanical characteristics of the type of steel. The accuracy of the model can be verified through the results of the non-linear analysis, taking into account the stiffness of the frames in each direction and the results obtained by applying the seismic records.

### 2.2. Applied Loads

When analyzing industrial warehouses and other structures, specific loads must be applied, such as overloads or wind loads that are of considerable magnitude whose effect can significantly affect the structure’s performance. In this study, however, the analysis considering these types of loads was only used to define the thicknesses of the coatings. Therefore, for the analyses presented from now on, only the loads transmitted due to their weight from all the coatings to their respective structural, façade, and roof elements will be considered.

Then, the weight of the panels is transferred in the form of a linear distributed load to each element of the shed, whether it is a siding or a beam. This weight approximation in the area to linear charge is made using the method of tributary areas. For this, all views of the warehouse are used, whether front, side, rear, and top, to divide the areas from the panels to the beams and laterals. For the dynamic analysis, the loads coming from the weights of the structural members, and the non-structural components, are transformed into masses. The overhead crane will carry a maximum load of 70 kN and a minimum load of 21.8 kN. The loads from the overhead crane are applied differently at each extreme position as if the overhead crane wwas stationary and directly on each of the frames oriented in the transverse direction.

### 2.3. Pushover Analysis

Nonlinear static analysis, also known as pushover analysis, is a calculation method for seismic design widely used in Latin America and the United States [5,46,47,48] and used in the European seismic code (EC8, 2004) [49]. The calculation method lies between static linear and dynamic nonlinear analysis. The pushover analysis seeks to bring the structure to collapse by applying incremental lateral loads to obtain the capacity curve of the structure (lateral displacement versus basal shear force) that allows assessing the structure’s performance according to the following criteria:

- Immediate Occupancy (IO): At this performance level, it is expected that the structure has a low level of damage, having mainly the same initial resistance and rigidity, and remains fully operational after the earthquake [41];
- Life Safety (LS): At this performance level, the structure is expected to suffer damage to its main structural components after the earthquake but preserve a margin of resistance and safety against a possible partial or total collapse so that human lives are not in threat [41];
- Collapse Prevention (CP): At this level of performance, the structure is expected to be significantly damaged and continue to support gravity loads without having a margin of resistance against a partial or total collapse [41].

The application of the static nonlinear analysis was conditioned by the existence of only one level of the steel structure, defined by the roof. Additionally, the stiffness provided by the roof panels does not allow for considering it acting as a rigid diaphragm. All this has required a previous analysis to determine the lateral stiffness of the frames in the transverse direction of the structure since the external frames are much more rigid than the internal frames. Once the stiffness of the frames was calculated, incremental forces were applied to the upper ends of the columns, with these incremental forces being proportional to the stiffness of each frame, ensuring a uniform lateral displacement, as recommended in previous works [26,27,50]. Once uniform displacements have been guaranteed, the control node chosen for these analyses has been the center of mass of the roof level (frame 4), given the regularity of the structure studied both in plan and elevation. In case the conventional pushover analysis procedure is applied, it is highly recommended to follow the recently published recommendations [23,24] that address procedures to improve the distribution of lateral forces and the choice of control nodes.

**Figure 4:**
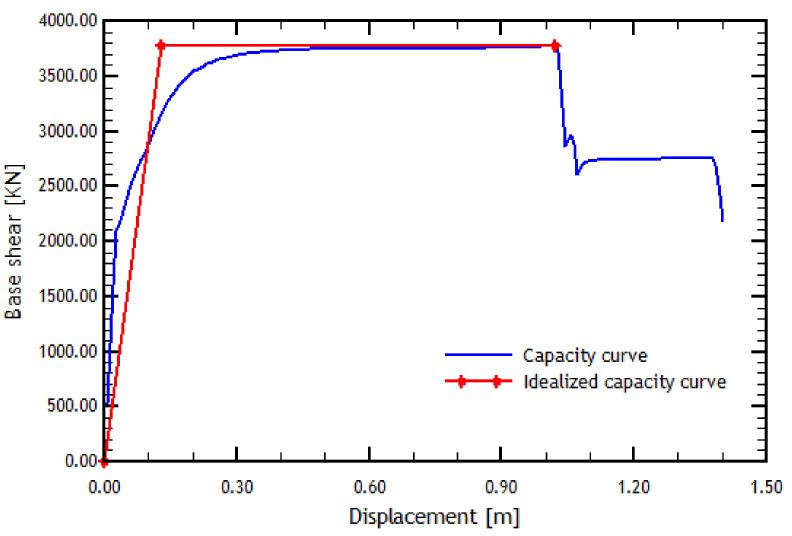
Analytical and idealized capacity curves in the longitudinal direction.

The capacity curve is idealized using a procedure in which the real and idealized curve energies are equalized [51]. Figure 4 shows an example of an analytical capacity curve and the idealized capacity curve, where it can also be noted that the idealized curve compensates for the areas for its construction [9].

In addition to having the pushover curves in the longitudinal and transverse directions, a pushover analysis will also be carried out in both directions considering the application of loads from an overhead crane, which will be arranged on the brackets defined in the structural model. The crane load cases included in the analyses are as follows:

Pushover analyses will be performed according to the longitudinal and transverse directions and also according to the consideration of the following two cases:

- Case 1: The maximum load of the crane is applied to the bracket of the column of the structural axis A, and the minimum load of the crane is applied to the bracket of the column of the structural axis D;
- Case 2: The minimum load of the crane is applied to the bracket of the column of the structural axis A, and the maximum load of the crane is applied to the bracket of the column of the structural axis D.

**Figure 5:**
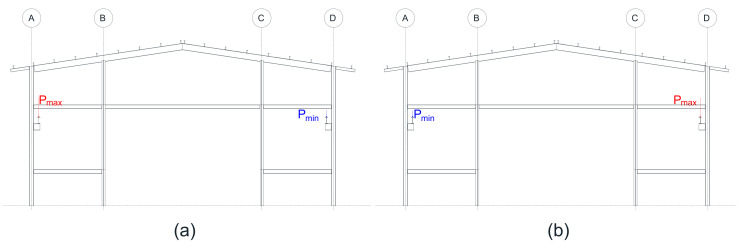
Consideration of overhead crane loads (**a**) Case 1 (**b**) Case 2. A, B, C and D are the structural axis.

The scheme of the crane loads applied is shown in Figure 5 for both cases.

### 2.4. N2 Method

**Figure 6:**
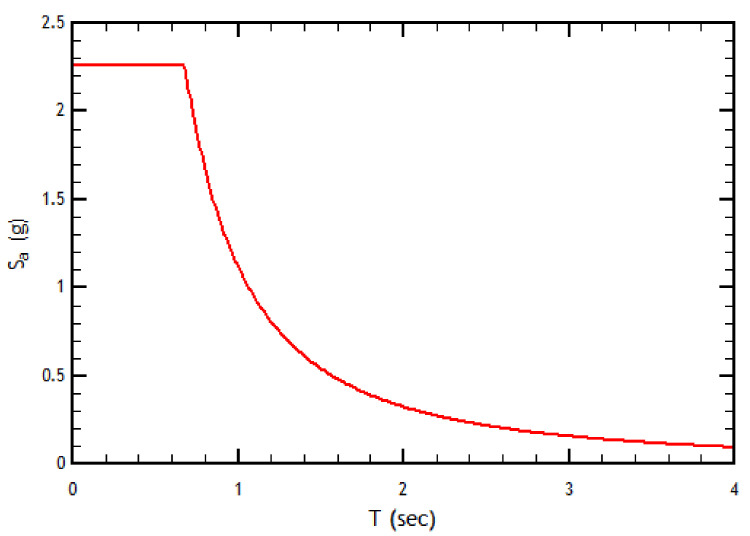
Design response spectrum according to the NCh2369 [40].

The N2 method, initially developed by Fajfar [52], is a method that requires the application of a nonlinear method based on two mathematical models (hence, the acronym N2) [53]. The N2 procedure has been extensively used to perform seismic response analysis of various structural typologies, as presented in [54,55]. The procedure consists of the following steps: (i) first, the stiffness, resistance, and ductility are determined by a nonlinear static analysis using a system of several degrees of freedom under the action of a monotonically increasing load distribution; (ii) then, it requiresdefining an equivalent system with a single degree of freedom whose characteristics are based on the base shear-top displacement relation defined in the nonlinear static analysis of the first step; (iii) from the nonlinear dynamic analysis of one degree of freedom, the maximum displacement (with its respective ductility) is determined, which can be done using inelastic spectra, as is the case of this investigation [4]. Therefore, the process considered in this work consists of the following steps:

1. Pushover analysis of the multi-degree-of-freedom model is performed to obtain the capacity or pushover curve. This procedure is carried out in this case before using the N2 method using the V2022 of SeismoStruct software [56];
2. The last curve is transformed to an equivalent pushover curve of a single degree of freedom using the following equations:

(1) $$F^{*}=\frac{F_{b}}{\Gamma_{1}}$$

(2) $$d^{*}=\frac{d_{n}}{\Gamma_{1}}$$

(3) $$\Gamma_{1}=\frac{\sum_{r}^{N}m_{r}\phi_{r,1}}{\sum_{r}^{N}m_{r}\phi_{r,1}^{2}}$$

where $F_{b}$ represents the base shear force, $d_{n}$ is the displacement of the control point, N is the number of stories, and m is the structure’s mass;

3. The equivalent period $T^{*}$ is graphically calculated using the slope of the elastic zone of the idealized curve;
4. The design spectrum is defined according to the NCh2369 code [40]. A series of steps allows defining the design acceleration spectrum [11], obtaining the graph depicted in Figure 6;

Then, the displacements are obtained using the calculated values and Equation (4).

(4) $$S_{de}{}^{*}=\frac{T^{2}}{4\pi^{2}}S_{ae}$$

where $S_{ae}$ corresponds to the acceleration of the design spectrum. From the $S_{de}$ versus $S_{ae}$ plot, the value of the breaking point of the curve, corresponding to a $S_{de}$ value, is defined as the $T_{c}$ value.

5. Finally, the graphs described in steps 2 and 4 are superimposed, and in case the initial zone slope of the pushover curve, and thus its prolongation, is before the break of the design spectrum ($T^{*}<T_{c}$), the point of intersection must be found and extended towards the horizontal axis to then calculate the ductility. Conversely, if the initial slope of the pushover curve is after the break of the design spectrum ($T^{*}>T_{c}$), the point of intersection has to be extended towards the horizontal axis to find the performance point.

### 2.5. Time–History Analysis

The time history analysis method consists of a mathematical model that uses hysteretic models to represent structures’ nonlinear behavior against seismic forces’ action. This method is used when a deeper study of seismic behavior is required since it represents more closely the structural behavior by using the inelastic properties of materials.

In a recent study, recommendations have been established for selecting near-field earthquake records to study the performance of steel structures [57]. Another study established the relationship between equivalent loads in industrial steel racks and dynamic actions induced by seismic records [58].

The ground motions used in this work correspond to records obtained from two destructive Chilean earthquakes. The seismic characteristics of these earthquakes and the recording stations are summarized in Table 2.

**Figure 7:**
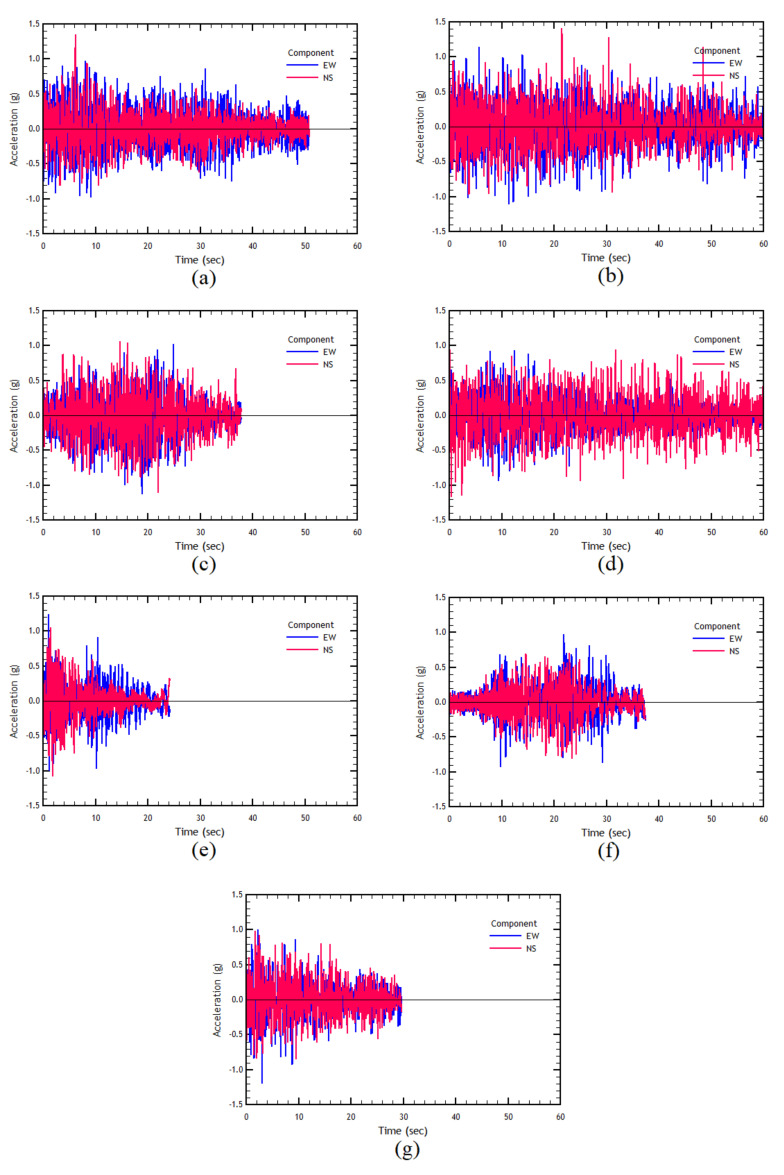
(**a**) Maule earthquake, Angol station record; (**b**) Maule earthquake, Concepcion station record; (**c**) Maule earthquake, Llolleo station record; (**d**) Maule earthquake, Constitucion station record; (**e**) Coquimbo earthquake, El Pedregal station record; (**f**) Coquimbo earthquake, San Esteban station record (**g**) Coquimbo earthquake, Tololo station record.

The earthquake records have been matched with the design spectrum shown in Figure 6 for a range of periods between 0.05 and 4.0 s. The duration of the records has been reduced by applying the 5% and 95% of the Arias Intensity [59]. The two components of the paired and trimmed records are shown in Figure 7, for the set of records used in this study.

For the analysis, various combinations of the selected ground-motion records are considered. First, each record component is applied separately in each direction (transverse and longitudinal), that is, in one analysis, the EW component is applied in the transverse direction and then applied in the *Y* direction in another analysis. The same procedure is considered for the NS component of the record and repeated subsequently for each seismic record.

In addition to combining components separately, a set of analyses were conducted considering both components simultaneously, each in a different direction. For example, in one case, the EW component is applied in the *Y* direction, and the NS component is applied in the transverse direction. In the second case, the record components are exchanged, leaving the component EW applied in the transverse direction, and the NS component applied in the longitudinal direction.

An interesting output from the time–history analyses of this study is the displacement of the columns’ top to monitor their state and behavior during and after the applied seismic loads. Once the relative displacement of the upper part to the base of the column is obtained and divided by the height of the column, the interstory drifts of each column of the structure are obtained. Using these interstory drift values and the ASCE-SEI 41 [41] standard, the seismic performance of the structure’s elements can be verified and compared to the three acceptance criteria: immediate occupancy (IO), life safety (LS), and collapse prevention (CP). Finally, the structural damage of each column is estimated. Based on this, an improvement of the most affected columns may be studied later.

## 3. Results 

### 3.1. Pushover Analysis

**Figure 8:**
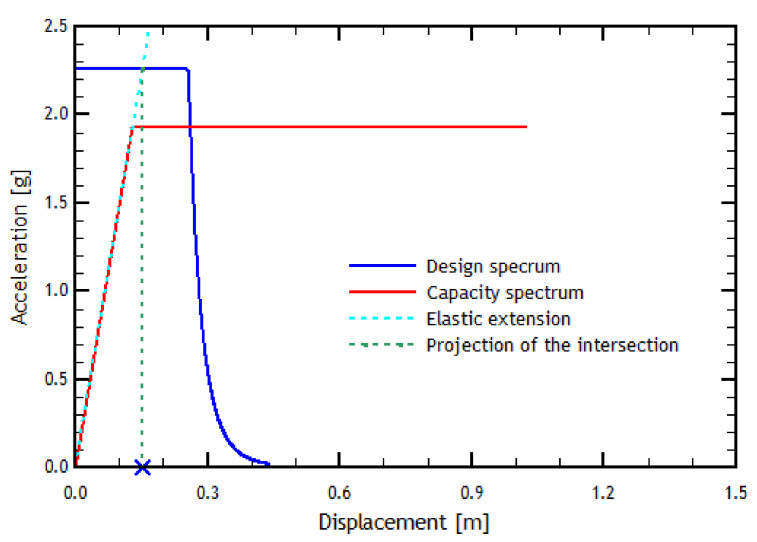
Determination of the performance point using the N2 method in the longitudinal direction. The blue cross represents the Performance Point displacement.

As mentioned earlier, a pushover analysis case without the effect of the overhead crane is considered. Additionally, the result of the pushover analyses must go hand in hand with the result of the N2 method due to software limitations. The results of the longitudinal direction analyses are presented in Figure 4 and Figure 8.

**Figure 9:**
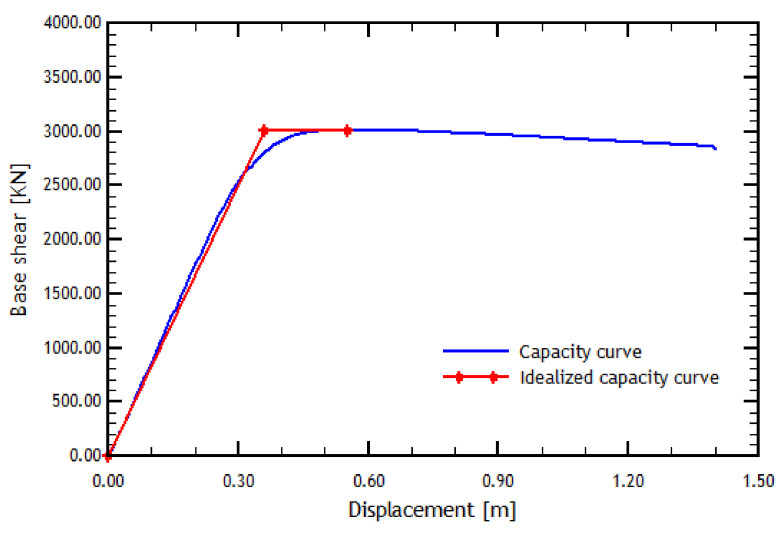
Capacity and idealized curve, transverse direction.

As seen in Figure 8, by prolonging the elastic linear zone of the pushover curve in the *Y* direction, it passes through the elastic zone of the design spectrum, which implies a demand for ductility of a considerable value for the reference structure. This result means there would be an inelastic demand in the *Y* direction, where the performance point corresponds to the *X*-axis value multiplied by the ductility value. Similarly, for the pushover analysis in the transverse direction, the results are shown in Figure 9.

The fundamental periods in each structure direction are required to apply the N2 method. For this, the periods of the modal analysis are obtained and presented in Table 3.

**Figure 10:**
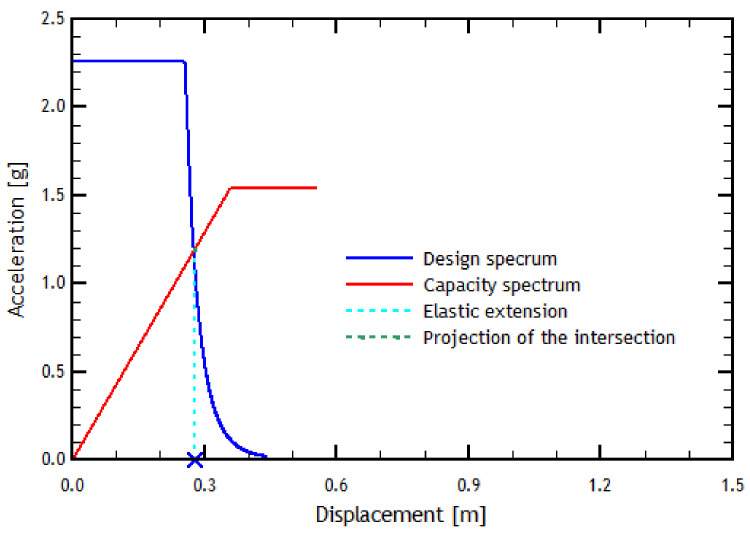
Determination of the performance point using the N2 method in the transverse direction. The blue cross represents the Performance Point displacement.

Figure 10 shows the results of the N2 method applied in the transverse direction. In this case, and unlike the longitudinal direction, the load imposed on the structure in the transverse direction causes the structure to respond within the elastic range. Therefore, there is eventually no ductility demand in the transverse direction, indicating that the structure behaves inelastically. This result means that the performance point is directly the displacement value of the intersection point of the curves, found by projecting the point on the *X*-axis.

Table 4 shows the values of ductility and performance points of the pushover analyses in the *X* and *Y* directions. These results are then used along with the load exerted by the overhead crane, which includes a vertical component equal to the total crane load plus a horizontal component equal to 10 percent of the vertical crane load. This load is applied in brackets arranged in each column. The load in each frame is considered separately and in two cases, one with a maximum load in the left column of the frame and the minimum load in the right column (case 1), and another with the maximum load in the right column of the frame and the minimum load in the left column (case 2). The results of the most significant cases in each direction of analysis shows a considerable number of cases (2 per frame).

For the analysis in the transverse direction, the pushover and the N2 method results are shown below for Frame 7-case 1 and Frame 6-case 2 cases. The cases for Frame 1 are considered for the *Y*-direction analysis-case 1 and Frame 7-case 2.

When analyzing the results in the longitudinal direction with the overhead crane action included, it can be noted that when placing the loads on the brackets of the frames, the effect of the crane does not vary depending on the frame in which it is located, since the frames in the *Y* direction are identical and therefore share the same structural characteristics. In addition, it is expected that when applying the loads in the outer frames (1 and 7), the analysis in the longitudinal direction would present its most significant effect since, being at the edges of the frames, the force opposed to the incremental load is less due because is not supported with the force that the interior frames would provide. Even so, in this direction, that effect is minimal since the difference in the value of the maximum shear between the external and internal frames in the *Y* direction is small compared to the maximum shear (10–20 kN). Table 5 presents the results and values of the ductility and displacements of performance points for the previous analyses.

Table 5 and Table 6 show the results of ductility and performance point displacement (P.P. displacement) calculated for different static nonlinear analyses, considering the crane load applied in the corresponding position of the frames from 1 to 7.

The results indicate that considering crane loads located in different positions produces differences in ductility of 0.8% and the displacement of the performance point of 0.07% in the transverse direction. In comparison, the differences in the longitudinal direction reach 0.05% and 0.3% for ductility and displacement of the performance point, respectively. Regarding the difference of both parameters when not considering the presence of crane loads, it reaches values of 0.6% and 0.04% for ductility and displacement of the performance point in the transverse direction, whereas for the longitudinal direction, not considering the crane loads introduces differences of 0.04% and 2.12% for ductility and displacement of the performance point, respectively. According to these results, the influence of considering crane loads on the results of the static nonlinear analysis can be considered negligible.

### 3.2. Time–History Analysis

**Figure 11:**
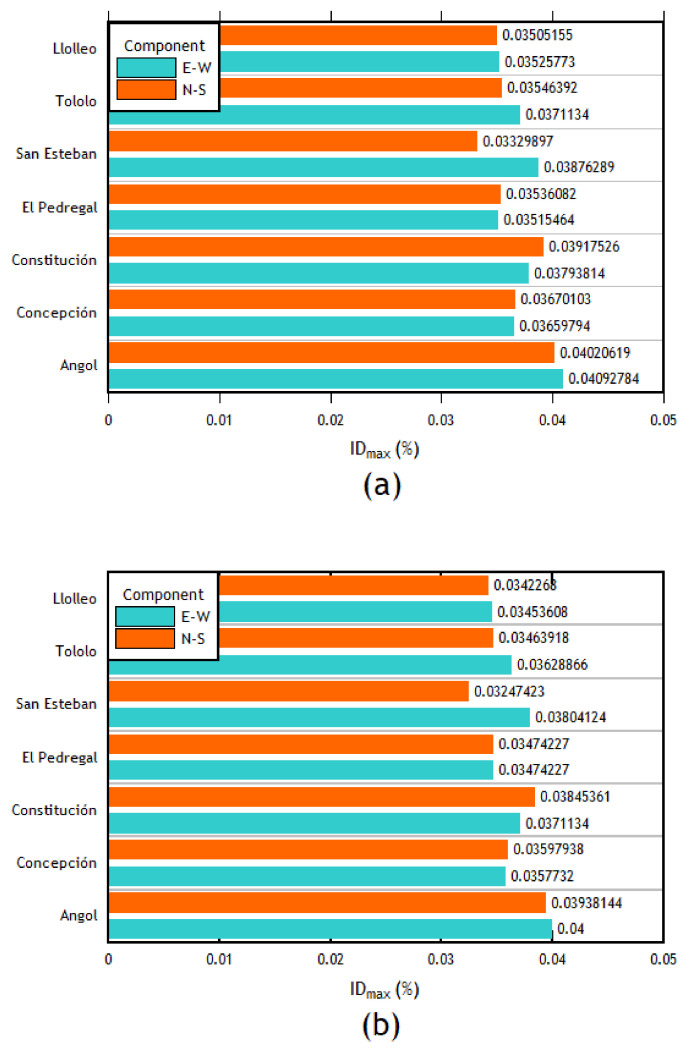
Interstory drifts of (**a**) the right column of frame 4 and (**b**) the right column of the frame 4 transverse direction.

The interstory drifts for each record are presented in Figure 11a. First, in the transverse direction, the column with the maximum interstory drift with almost all the records in both components (EW and NS) corresponds to the right column of frame 4. When analyzing the results in the transverse direction, it can be expected to be the most affected column with the most significant displacements since it corresponds to the frame right in the middle of the structure. Followed by the right column of frame 4, the column with the most significant deviations corresponds to the right column of frame 3. As can be seen, it also belongs to the central columns of the structure. Therefore, in the transverse direction of analysis, this behavior is expected. The interstory drifts of each component applied in the transverse direction for this column are shown in Figure 11b.

**Figure 12:**
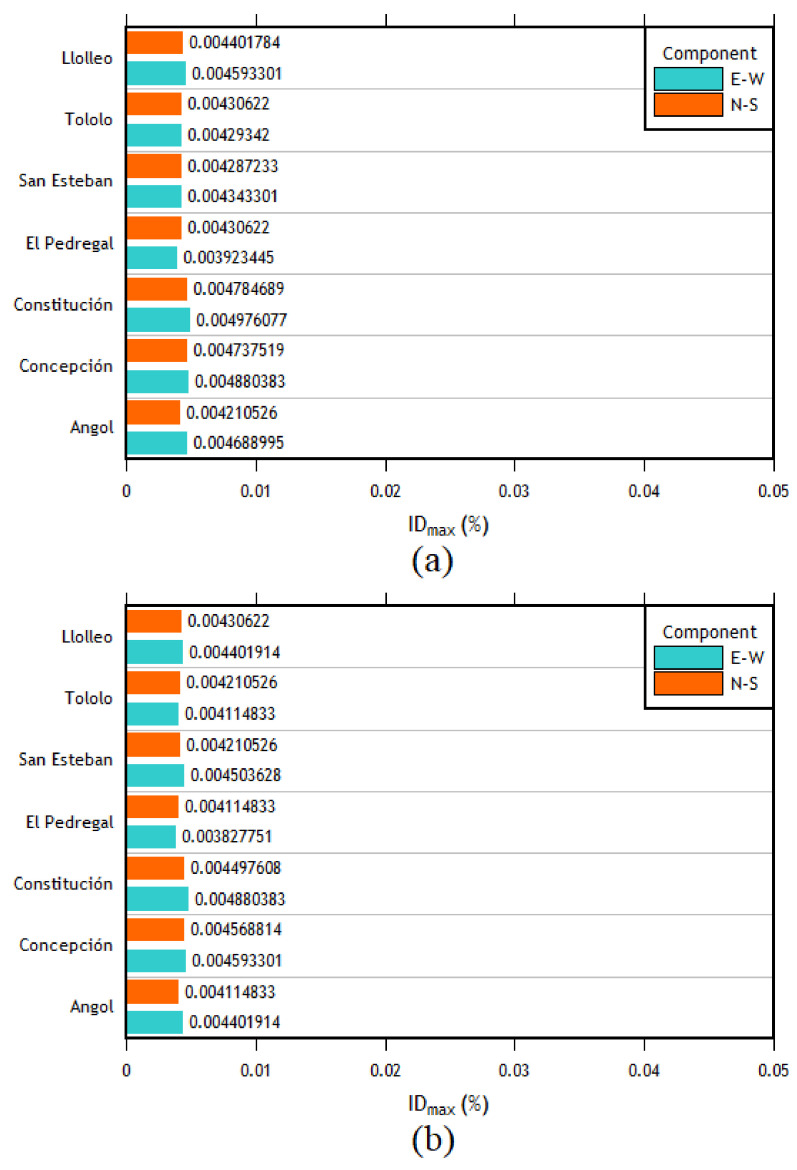
(**a**) Left center column interstory drifts of the outer frames, longitudinal direction. (**b**) Right center column interstory drifts of the outer frames, longitudinal direction.

The analyses in the longitudinal direction show that the column with the highest interstory drift values for most of the imposed records was the central left column of the outer frames. Given that there are no intermediate columns in this frame, the first and the last frames present the same interstory drifts. Therefore, the interstory drifts of the two central columns of the outer frames (1 and 7) are the most important (see Figure 12a,b).

**Figure 13:**
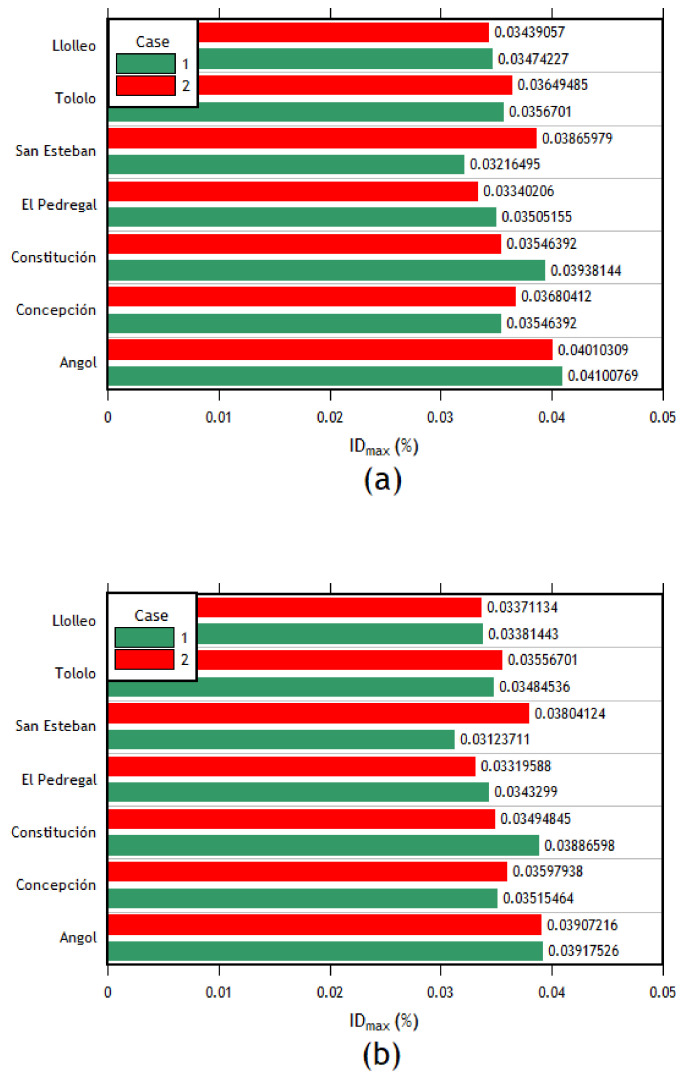
Interstory drifts of the (**a**) right column of frame 4 and (**b**) right column of frame 3, cases 1 and 2.

After analyzing the results of each direction separately, the combination of cases was carried out. The following cases were defined: for case 1, the EW component was applied in the longitudinal direction, and the NS component was applied in the transverse direction; in case 2, the EW component was applied in the transverse direction, whereas the NS component was applied in the longitudinal direction. The results are presented in Figure 13a,b. As can be seen, the right column of frame 4 is the most important, followed by the right column of frame 3 in the transverse direction. This trend is similar to the one observed in the individual analyses.

The difference in the transverse displacements of the left column of axis 4 resulting from the analysis without considering the crane loads for cases 1 and 2 is 2.34% and 1.11%, respectively. Similarly, the difference in the longitudinal displacements of the left central column of axis 1 for cases 1 and 2 is 0.76% and 2.96%, respectively. It can be observed that, just like the results obtained by static nonlinear analyses, the results of nonlinear dynamic analyses are minimally affected when considering the crane loads.

**Figure 14:**
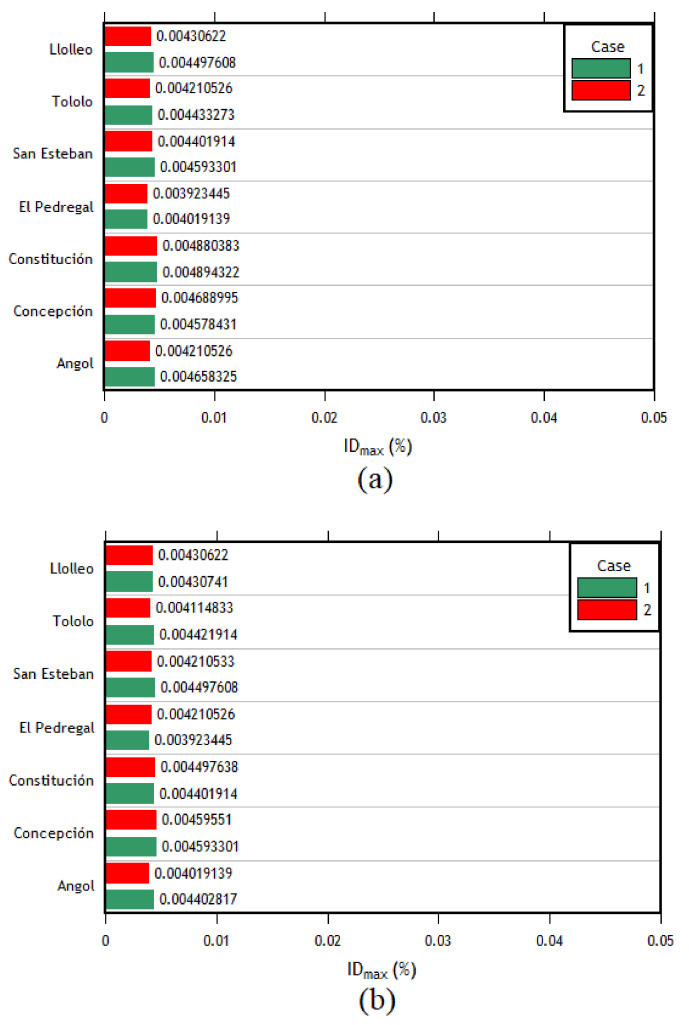
(**a**) Interstory drifts of the left central column of the external frames, cases 1 and 2 in the longitudinal direction; (**b**) Interstory drifts of the right central column of the external frames, cases 1 and 2 in the longitudinal direction.

The same behavior trend is presented in the individual analysis for the results in the longitudinal direction. This trend means that the most requested columns are the central columns of the outer frames, and both columns of frames 1 and 7 present the same interstory drifts. The results of these columns are presented in Figure 14. Finally, after finding the interstory drifts for all the columns, the maximum interstory drift for each column between each combination of directions for each seismic record was obtained. Later, the average of these maximum values was computed, obtaining a single interstory drift value per column. Then, using the method described in the ASCE 41 [41] standard, the seismic acceptance criteria for each column were calculated to assess their condition after the earthquake forces were applied. The results are presented in Table 7.

## 4. Discussion

The pushover analyses showed that the reference structure has greater ductility in the transverse direction, according to the pushover curves depicted in Figure 4 and Figure 9. From this, it can be inferred that the structure in the longitudinal direction collapsed after one meter of displacement. Although the structure in the transverse direction loses resistance, it is much more ductile since it reaches a more significant displacement without reaching the failure. However, since this displacement is considerably high, the reference structure shows good global behavior. It is also found that the transition from elastic to plastic behavior in the capacity curve in the longitudinal direction occurs for a lesser displacement than in the transverse direction. This trend is because the period of the structure in the transverse direction is more significant than in the longitudinal direction and is evidenced by an initial zone of the curve with a considerably greater slope.

Following the pushover analysis in each direction, the load exerted by the overhead crane that runs over the length of the warehouse is included. This difference is marginal when comparing the results with and without loads of the overhead crane. In addition, the values of the internal forces resulting from the elements are similar.

In addition to the pushover analyses, an analysis using the N2 method was investigated. The analysis in the longitudinal direction shows that the result of the N2 method indicates that the structure is behaving nonlinearly with a small ductility demand. In the transverse direction, the N2 method indicates that the structure is within the elastic range and has a large displacement, obtaining that the difference in displacement between both directions is approximately half. This result can be verified with the ductility values since, in the longitudinal direction, the ductility value is more significant than 1. In the transverse direction, however, it is less than 1 (see Table 8). The fact that the structure in the transverse direction has a longer period than in the longitudinal direction causes the capacity spectrum to move to the right, whereby the demand is related to the capacity for high periods. This result means that the structure in this direction would behave elastically. In contrast, for the longitudinal direction, the opposite happens since everything is related in the zone of low periods. Therefore, the structure’s capacity demand occurs in an inelastic zone (see Figure 10).

The time–history analysis allowed calculating the interstory drifts of each column for each case of analysis. Initially, an individual analysis was carried out in each direction to carry out an analysis with combined cases later but in each direction (*X* and *Y*). Then, having the value of the interstory drifts applying each seismic record for each combination of components in each direction of analysis only used the highest interstory drift value between the same direction of each earthquake in order to finally obtain an average of the interstory drifts of each column for each direction of analysis. With this value, it can be compared with the interstory drift values corresponding to each acceptance criteria (IO, LS, CP) which were obtained using the ASCEI/SEI 41 [41]. The order of the maximum interstory drifts in the transverse direction is between 0.0124 and 0.0373; in the longitudinal direction, it varies between 0.0032 and 0.0046.

As can be seen, most of the columns are within the acceptance criteria for life safety (LS) for the transverse direction. However, some columns in the center of the structure are within the acceptance criteria for collapse prevention. This observation agrees with the results since, in the time–history analyses, the most affected columns (in the transverse direction) were the central columns (frames 3, 4, and 5). This pattern is expected due to their configuration compared to the external frames. In addition, taking into account the values of interstory drifts and the difference in magnitude between the *x* and longitudinal directions, it was expected that the performance in the longitudinal direction would be better, which is reflected by the fact that all the columns are within the immediate occupancy (IO) performance level. This difference in the magnitude of interstory drifts is mainly due to the structuring that exists in each direction since, in the longitudinal direction, the structure has braced frames.

A contradiction can be found in the results obtained by the time–history, pushover, and N2 methods. This is because, in the N2 method, results indicate that the structure in the transverse direction behaves and responds within the elastic range. In contrast, in the longitudinal direction, the structure responds nonlinearly. There is also a proportion of the response between each direction of analysis, approximately half. This contradicts what was obtained by the time–history method since the results of this method indicate that the most requested analysis direction (due to the interstory drifts obtained, in addition to the final criteria of each column) is the transverse direction and not the longitudinal direction, as with the N2 method. In addition, the proportion of the results of the maximum interstory drifts obtained in *X* is 10 times greater than those obtained in the longitudinal direction, not ½ as indicated by the N2 method. This means that the N2 method for this type of structure is specifically not suitable because of the kind of structure.

## 5. Conclusions

When globally analyzing the results, the pushover method and the time–history method are reasonably consistent since they show that the transverse direction is more ductile, agreeing with the type of structure of the warehouse frames.

The maximum differences in ductility and performance point displacements determined by the static nonlinear analysis with and without considering the crane loads reach 2.12% and 0.60%, respectively. As for the results of the nonlinear dynamic analyses, the calculated displacements without considering the crane loads present a maximum difference of 2.34% and 2.96% in the transverse and longitudinal directions, respectively. According to these results, it is concluded that for the case study, including crane loads has little effect on the results.

Regarding the time–history method, the results of the analyses in the transverse direction present greater interstory drifts in the internal frames of the structure. This result agrees with what was expected since these frames, being further away from those that are more rigid than the extremes, should tend to a more significant displacement, something that was reflected in the results of the investigation. Something similar occurred in the pushover analysis results since the direction that presented the most significant displacements was the transverse direction, even not breaking as did the longitudinal direction.

The N2 method fails to adequately capture the behavior of the structure since its results are contradictory when compared to those obtained with time–history analyses.

The results of the time–history analyses show that the structure is affected differently in the main directions of the structure, which is reflected in the results of the acceptance criteria and indicates that there are several columns that, for the analysis direction *x*, reach different limit states, including the Limit State of Collapse Prevention (CP). However, in the braced frames, which correspond to the analysis direction *Y*, all the columns have a performance that is located within the Limit State of immediate occupancy (IO). This diversity of performance must be considered if it is sought to guarantee an acceptable overall performance, to protect non-structural elements and especially the contents of the house.

For future research, it is suggested to study the behavior of this type of structure by varying the geometry of the shed, considering asymmetric structures or with different span lengths that produce different loads on the elements and confirm the finding of this research about the influence of the crane load on the results.

## Figures and Tables

**Table 1:** Components’ cross sections of the structure.

Component	Cross Section
Columns	HEB 300
Central columns	HEB 220
Rafters	IPE 450
Central beams	IPE 270
Bracings	CAE 50 × 50
Purlins	IPE 200
Cantilever	IPE 450

**Table 2:** Ground motion suite summary.

Station	Earthquake	Magnitude (Mw)	Depth (km)	Component	PGA (g)
Angol	Maule 2010	8.8	30.1	EW	0.681
				NS	0.928
Concepción	Maule 2010	8.8	30.1	EW	0.402
				NS	0.398
Llolleo	Maule 2010	8.8	30.1	EW	0.319
				NS	0.702
Constitucion	Maule 2010	8.8	30.1	EW	0.552
				NS	0.352
El Pedregal	Coquimbo 2015	8.4	23.3	EW	0.350
				NS	0.290
San Esteban	Coquimbo 2015	8.4	23.3	EW	0.268
				NS	0.182
Tololo	Coquimbo 2015	8.4	23.3	EW	0.240
				NS	0.340

**Table 3:** Predominant modes of vibration in each direction of the structure.

Direction	Period (s)	Modal Participation Factor	Modal Mass (Tonne)	% of Participative Mass
Transverse	0.543	8.130	66.099	74.592
Longitudinal	0.225	8.805	77.536	87.499

**Table 4:** Results of the pushover analysis.

	Direction *X*	Direction *Y*
Ductility	0.7717	1.1679
Performance point displacement [m]	0.2772	0.1776

**Table 5:** Results of the pushover analysis including the load of the overhead crane.

Frame	Case	Ductility	P.P. Displacement (m)
1	1	0.7719	0.2773
	2	0.7759	0.2771
2	1	0.7711	0.2773
	2	0.7756	0.2771
3	1	0.7706	0.2773
	2	0.7736	0.2771
4	1	0.7707	0.2773
	2	0.7761	0.2771
5	1	0.7734	0.2773
	2	0.7763	0.2771
6	1	0.7715	0.2773
	2	0.7742	0.2771
7	1	0.7715	0.2773
	2	0.7761	0.2771

**Table 6:** Results of the pushover analysis including the load of the overhead crane.

Frame	Case	Ductility	P.P. Displacement (m)
1	1	1.1733	0.1736
	2	1.1733	0.1736
2	1	1.1737	0.1734
	2	1.1737	0.1734
3	1	1.1738	0.1732
	2	1.1738	0.1732
4	1	1.1739	0.1733
	2	1.1739	0.1733
5	1	1.1739	0.1733
	2	1.1739	0.1733
6	1	1.1737	0.1732
	2	1.1737	0.1732
7	1	1.1733	0.1737
	2	1.1733	0.1737

**Table 7:** Results of acceptance criteria for each column, using the average of the maximum interstory drifts for each one.

Frame	Column	Interstory Drifts *Y* (avg.)	Interstory Drifts *X* (avg.)	IO	LS	CP
1	Ext. right	0.0036	0.0134	0.0089	0.0313	0.0417
	Int. right	0.0033	0.0135	0.0073	0.0325	0.0434
	Int. left	0.0046	0.0125	0.0212	0.0553	0.0737
	Ext. left	0.0044	0.0126	0.0140	0.0555	0.0741
2	Left	0.0036	0.0153	0.0086	0.0303	0.0404
	Right	0.0033	0.0168	0.0086	0.0303	0.0404
3	Left	0.0035	0.0339	0.0083	0.0293	0.0390
	Right	0.0032	0.0365	0.0083	0.0293	0.0390
4	Left	0.0035	0.0345	0.0083	0.0292	0.0390
	Right	0.0032	0.0373	0.0083	0.0292	0.0390
5	Left	0.0035	0.0339	0.0083	0.0293	0.0390
	Right	0.0032	0.0365	0.0083	0.0293	0.0390
6	Left	0.0035	0.0152	0.0086	0.0303	0.0404
	Right	0.0033	0.0167	0.0086	0.0303	0.0404
7	Ext. right	0.0035	0.0133	0.0089	0.0313	0.0417
	Int. right	0.0032	0.0135	0.0089	0.0313	0.0417
	Int. left	0.0045	0.0124	0.0212	0.0553	0.0737
	Ext. left	0.0044	0.0126	0.0212	0.0553	0.0737

**Table 8:** Acceptance criteria for each column for each direction of analysis.

Frame	Column	Acceptance Criteria	
		Direction *X*	Direction *Y*
1	Left outer	LS	IO
	Right outer	LS	IO
	Left inner	IO	IO
	Right inner	IO	IO
2	Left	LS	IO
	Right	LS	IO
3	Left	CP	IO
	Right	CP	IO

## Data Availability

Not applicable.
